# Somatic CTG•CAG repeat instability in a mouse model for myotonic dystrophy type 1 is associated with changes in cell nuclearity and DNA ploidy

**DOI:** 10.1186/1471-2199-8-61

**Published:** 2007-07-23

**Authors:** Walther JAA van den Broek, Derick G Wansink, Bé Wieringa

**Affiliations:** 1 Department of Cell Biology, Nijmegen Centre for Molecular Life Sciences, Radboud University Nijmegen Medical Centre, The Netherlands

## Abstract

**Background:**

Trinucleotide instability is a hallmark of degenerative neurological diseases like Huntington's disease, some forms of spinocerebellar ataxia and myotonic dystrophy type 1 (DM1). To investigate the effect of cell type and cell state on the behavior of the DM1 CTG•CAG repeat, we studied a knock-in mouse model for DM1 at different time points during ageing and followed how repeat fate in cells from liver and pancreas is associated with polyploidization and changes in nuclearity after the onset of terminal differentiation.

**Results:**

After separation of liver hepatocytes and pancreatic acinar cells in pools with 2n, 4n or 8n DNA, we analyzed CTG•CAG repeat length variation by resolving PCR products on an automated PAGE system. We observed that somatic CTG•CAG repeat expansion in our DM1 mouse model occurred almost uniquely in the fraction of cells with high cell nuclearity and DNA ploidy and aggravated with aging.

**Conclusion:**

Our findings suggest that post-replicative and terminal-differentiation events, coupled to changes in cellular DNA content, form a preconditional state that influences the control of DNA repair or recombination events involved in trinucleotide expansion in liver hepatocytes and pancreatic acinar cells.

## Background

Instability of CTG•CAG trinucleotide repeats forms an important class of mutations on an ever-growing list of inheritable degenerative neurological diseases, including Huntington's disease, several spinocerebellar ataxias (SCAs 1, 2, 3, 6, 7, 8, 12 and 17) and myotonic dystrophy type 1 (DM1) [1-3]. Depending on disease type, pathogenic mechanisms may vary, but all disorders have in common that the transition to illness appears to be a cell-intrinsic process, which is initiated by intergenerational or somatic repeat expansion or both. Although substantial progress has been made by the study of expansion behavior and associated pathophysiological events in repeat-containing bacterial and yeast model systems [4], translation of findings to the mammalian system is not easy and there is still disagreement over the contribution to CTG•CAG repeat expansion by new strand synthesis during DNA replication or DNA repair and recombination in cycling or quiescent cells for the various diseases. Also effects of cell-type and cell-state during development, adulthood and aging are generally not well understood. Yet, exactly this knowledge is needed to aid in the development of therapeutic modalities.

Since the discovery of its molecular defect, DM1 has been an archetypal case within the group of unstable trinucleotide diseases. In DM1 both progenitor CTG•CAG repeat length and somatic expansion determine age of onset and severity of disease, which is characterized by muscular dystrophy and myotonia in combination with a highly variable manifestation of features like cataract, heart conduction defects, insulin insensitivity and cognitive impairment. The DM1 mutation is an expansion of a CTG•CAG repeat in the 3' UTR of the *DMPK *gene on chromosome 19. Once DM1 alleles are in the disease-associated size range (>50 CTG•CAG repeats), repeat tracts become dramatically unstable. Intergenerational mutation rates may be almost 100% per generation, with a tendency towards further repeat gains [5-7]. The DM1 repeat is also somatically unstable and extensive instability in a wide range of human tissues is what makes DM1 a truly unique trinucleotide disorder [5,8,9].

Somatic expansion is mediated by multiple small length changes in a highly deterministic process which is clearly age-dependent, with longer average DM1 repeat length and broader ranges of variability observed in older patients [7,9]. We know that somatic expansions accumulate in both proliferating and post-mitotic tissues, suggesting that expansion is independent of cell division [8,10]. Furthermore, the rate of instability of the CTG•CAG repeat in DM1 is correlated to repeat length and is probably due to an increased ability of the repeat sequence to form aberrant DNA structures, such as slipped strand structures with out-of-register alignment of the complementary strands, cruciforms or intramolecular triplex and quadruplex structures with single-stranded regions [4]. Failure in the processing of these structures, a cascade of reactions in which mismatch repair proteins – e.g., MSH2, MSH3, PMS2 or MLH1 – likely play a role, may underlie mutagenic instability [11-14]. Aberrant DNA break repair induced by these structures may also be at the basis of instability [15]. In addition, c*is*-acting factors such as nearby replication origins or CpG islands may influence instability [3,16].

We here report on an analysis towards the role of cell type and cell stage in CTG•CAG instability in different tissues of a mouse knock-in DM1 model, where a human (CTG•CAG)_n _repeat segment (n ~ 110) replaces the cognate 3' segment of the endogenous mouse *Dmpk *gene [13]. We concentrated on tissues that continuously divide and tissues with non-cycling cells that undergo terminal maturation early in life or during aging.

## Results

Our group has developed a knock-in mouse model in which somatic expansion of the DM1 CTG•CAG tract is faithfully reproduced. In this animal model a short segment of human chromosomal DNA spanning the exon 13–15 region of the *DMPK *gene of a DM1 patient with an abnormal (CTG•CAG)_84 _repeat replaces the exact cognate segment of the endogenous mouse *Dmpk *gene. Hence, this places chromatin embedding of the repeat element and production of repeat containing DMPK mRNA under "natural" host control. Study of this model revealed that the mutability of the repeat is age-dependent and activity of the mismatch repair protein Msh3 (usually found associated in the Msh2/Msh3 MutSβ repair complex) is important for repeat expansion, whereas the Msh6 protein (found in association with Msh2 in the MutSα complex) appears dispensable [13].

**Figure 1:**
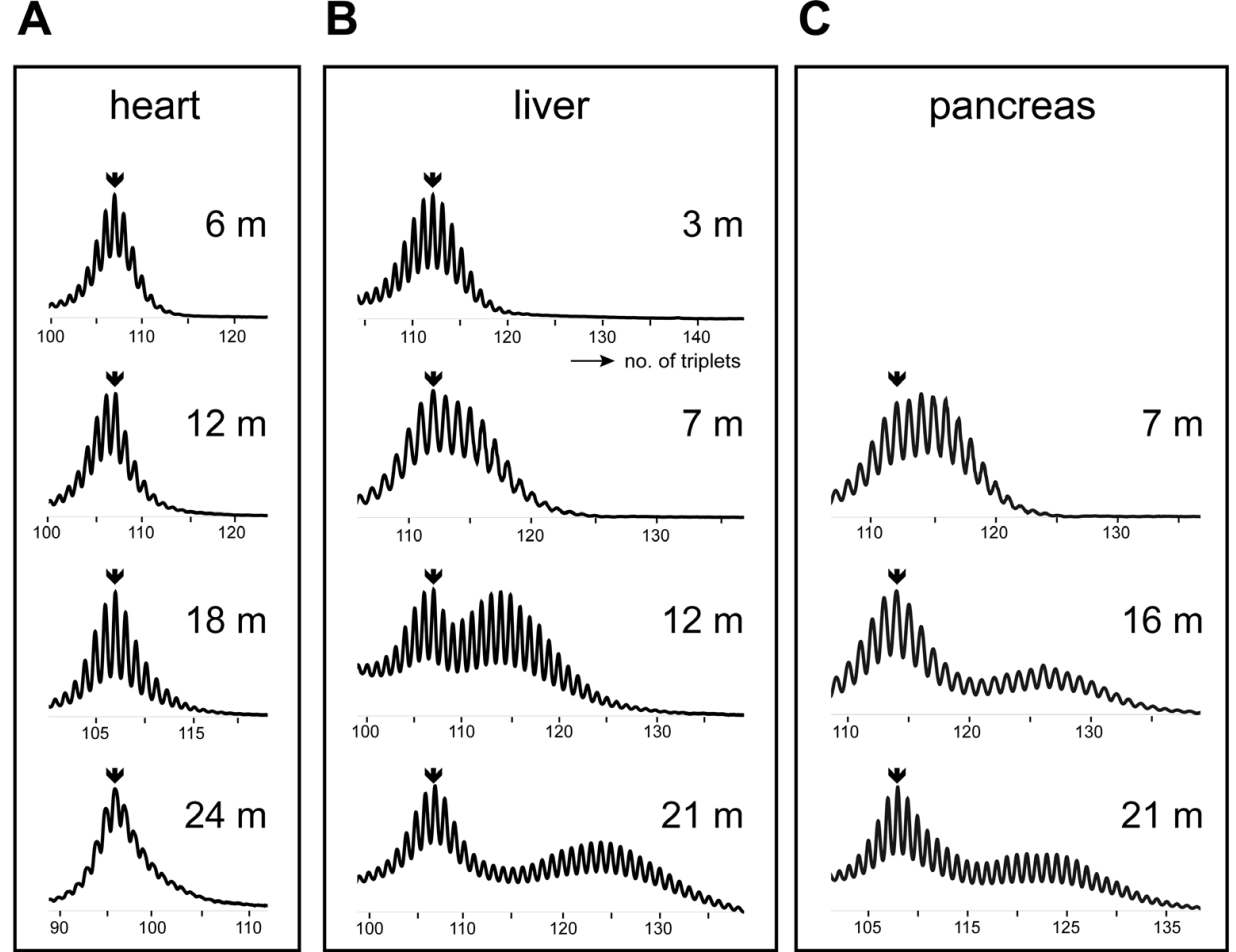
**Effects of aging on the somatic CTG•CAG repeat length distribution in heart, liver and pancreas**. Typical CTG•CAG length profiles of genomic DNA isolated from heart (A), liver (B) or pancreas (C) of a knock-in mouse model carrying a humanized DMPK gene with a (CTG•CAG)_n _repeat [13]. Arrows indicate the position of the progenitor allele determined from analysis of tail DNA at time of weaning. Note the slow but uniform broadening of the profile with slight shift towards greater repeat lengths upon aging in heart, in contrast to the bimodal repeat length distribution in liver and pancreas.

In this model, we now analyzed the cell-type and cell-stage dependence of CTG•CAG instability in different tissues in more detail. Analysis of PCR-amplified DMPK repeat segments originating from tissues with a moderate to low fraction of cycling cells like skin, bone marrow or heart showed a typical Gaussian distribution of allele sizes, slowly expanding to greater average length during aging (Fig. 1A). In contrast, tissues with a rather high repeat expansion rate, like kidney and stomach, displayed a significant broadening of the profile (see data presented in [13]), while liver and pancreas displayed a typical bimodal distribution profile (Fig. 1BC). Typically, after about six months of age the fraction of cells with no or moderately expanded alleles remained constant, but alleles in the already expanded pool of cells continued to increase in length. As the bimodal profile in liver and pancreas became increasingly pronounced with age, we considered effects of terminal differentiation and aging the best explanation for this phenomenon, rather than cell-type differences or tissue heterogeneity. Polyploidization, which is the state of acquiring a greater than diploid content of DNA, is one of few distinct events that mark physiologically normal late differentiation and is a distinguishing feature of liver hepatocytes and pancreatic acinar cells [17-19]. We therefore decided to concentrate on these cell types and analyze whether changes in cell nuclearity, hyperploidy or G2 state – or any state where two identical chromosome copies of the expanded CTG•CAG tract would coexist in one cell – could selectively affect repeat hypermutability via effects on homology-directed DNA recombination or repair.

**Figure 2:**
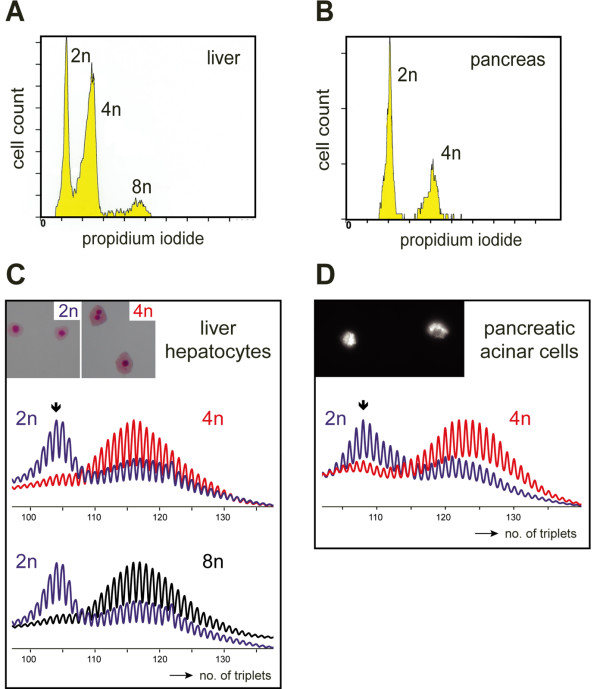
**Polyploid cells in liver and pancreas display enhanced somatic instability of the CTG•CAG repeat**. (A, B) Liver hepatocytes from a 17-month-old mouse and pancreatic acinar cells from a 21-month-old mouse were isolated, fixed, stained with propidium iodide and FACS purified based on DNA content. Preparative sorting profiles based on PI fluorescence intensity are shown. All 2n hepatocytes were mononuclear, whereas ~80% cells in the 4n and 8n pools of hepatocytes were binuclear (C, inset; typical examples of cells in the sorted 2n and 4n cell pools stained with Giemsa, are shown). Note that pancreas contained only diploid and tetraploid cells (a fluorescent PAS staining of zymogen inclusions established the identity of acinar cells (D, inset)). (C, D) CTG•CAG length profiles of hepatocytes and acinar cells demonstrated much larger average repeat length and almost complete absence of progenitor alleles in cells with 4n DNA (red; liver hepatocytes and acinar cells) and 8n DNA (black; liver hepatocytes only). In the 2n pools obtained from liver and pancreas (blue) only a small percentage of cells contained expanded repeats. Arrows indicate the position of the progenitor allele determined from analysis of tail DNA at time of weaning.

Hepatocytes constitute approximately 60–70% of liver cells and up to 90% of liver mass [17,18]. We employed a two-step collagenase perfusion protocol to disperse liver into single hepatocytes and non-parenchymal cells [20] (see Methods). Single hepatocytes were isolated and FACS fractionation was employed to sort the cell suspension in pools of 2n (diploid, mononuclear), 4n or 8n cells (both populations contained mononuclear and binuclear cells; collectively called polyploid) (Table 1; Fig. 2A,C inset). By analyzing the length distribution of PCR products from the repeat area on PAGE gels, and comparing peak areas in typical profiles as shown in Fig. 2C, we estimate that over 95% of hepatocytes in the 4 and the 8n cell pools harbored large expanded repeat alleles, whereas the majority of cells in the diploid cell pool carried repeats with progenitor allele size or only marginal length expansions (Fig. 2C). Comparison of different animals showed that less than 15–20% of the cells in the diploid pool harbored expansions and the percentage of expanded cells seemed to drop further with age. The typical overrepresentation of mutant alleles in the 4n and 8n cell pools was already apparent around six months of age when the polyploidization process had stopped, but the average repeat size increased further during aging (Table 1, and see data in [13]). As a cautionary note, we would like to stress here that the procedures used for liberation of single dispersed cells from tissue and the ploidy-sorting will not yield absolutely pure populations as cell aggregation and shearing cannot be avoided completely, and also cell cycle state (i.e., G0 or G2 phase) may affect the sorting efficiency. Yet, it is striking that expansion fate in the ≥4n pools always appeared much more homogeneous than that in the 2n pools, where 10–20% of cells appeared expanded. Importantly, the time course of genesis of 4n and 8n liver cells, and hence the postnatal development and maintenance of the polyploidization profile in our CTG•CAG knock-in lineage occurred as in wild type mice and was essentially completed within the first four months of life. Approximately 50% of all liver cells became tetraploid-octaploid, with a 20% versus 80% ratio between mono- and binuclearity (Table 1, data not shown). We thus may assume that the end-stage of differentiation in liver of our model is reached via a physiologically normal and step-wise process, with creation of binuclear cells preceding the formation of mononuclear tetraploid cells, as has been described in detail for rat liver [17].

**Table 1:** Correlation between age, somatic expansion, ploidy and nuclearity in DM1 knock-in mice

Age*	1 – 3 months			12 – 25 months		
Somatic Expansion	no			yes		

Ploidy	2n	4n	8n	2n	4n	8n
	54 ± 9%	45 ± 8%	2 ± 2%	45 ± 5%	43 ± 3%	13 ± 3%
Nuclearity	100% mononuclear	80% binuclear	n.d.	100% mononuclear	80% binuclear	80% binuclear

* n = 5 for 1–3 months-old mice and n = 8 for 12–25 months-old mice; n.d., not determined

**Figure 3:**
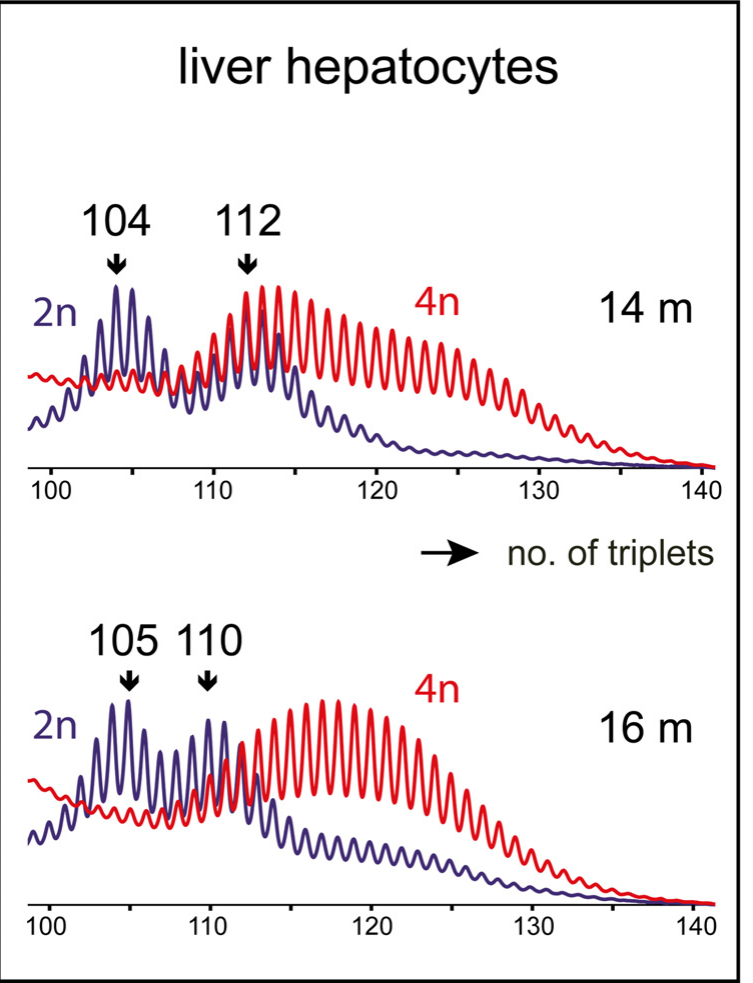
**Somatic instability of CTG•CAG repeats in liver hepatocytes of mice containing two humanized DMPK alleles (compound homozygotes)**. Hepatocytes were isolated from a 14-month-old compound homozygous (CTG•CAG)_104_/(CTG•CAG)_112 _and a 16-month-old (CTG•CTG)_105_/(CTG•CAG)_110 _mouse and sorted in 2n and 4n cells. PCR profiles of genomic DNA show that in both mice the average CTG•CAG repeat length in the pool of 4n cells (red) is much larger than in the 2n cell pool (blue) and shifted to larger length for both progenitor alleles. Arrows indicate the positions of the two progenitor (CTG•CAG)_n _alleles in tail DNA at time of weaning.

To study whether bi-allelic presence of expanded CTG•CAG repeats could further alter repeat mutability in liver – and vice versa, whether repeat dosage would affect liver cell polyploidization – we also intercrossed animals of our CTG•CAG knock-in lineage and followed repeat fate during aging of homozygous animals with (CTG•CAG)_104_/(CTG•CAG)_112 _or (CTG•CAG)_105_/(CTG•CAG)_110 _alleles. No effects of compound homozygosity were observed and repeat size profiles developed similarly in 2n, 4n or 8n hepatocyte pools from monoallelic (CTG•CAG)_110 _and compound homozygous (bi-allelic mutant) (CTG•CAG)_104_/(CTG•CAG)_112 _or (CTG•CAG)_105_/(CTG•CAG)_110 _mice during aging (Fig. 3). Also ploidy development occurred as in heterozygous or wild type animals.

Finally, to examine whether the apparent association between trinucleotide repeat expansion ability and polyploidization was more general in nature and occurred in other cell types as well, we monitored repeat mutation frequency also in pancreatic cells. In Figure 2B we show gel mobility analyses on PCR products from 2n and 4n pancreatic acinar cells collected after FACS analysis. Virtually all cells (>99%) in these preparations had retained typical acinar cell attributes including zymogen granules (Fig. 2D, inset). Notably, again a very high proportion of 2n cells (estimated fraction >90%) yielded signals in the original CTG•CAG size range, whereas alleles in the 4n acinar cells almost all were of higher length (Fig. 2D; less than 5% of 4n acinar cells contained alleles in the (CTG•CAG)_108 _range).

## Discussion and conclusion

In conclusion, although our data are of associative nature, our observations strongly suggest that polyploidization is not a condition that is directly involved in the expansion process per se, but merely establishes a cell state that must be reached before repeat expansion can be promoted later in life. If this cell state – considered the end-state of differentiation – is reached gradually and late in life of diploid cells, the ability to expand may even become manifest before the actual transition to multinuclearity or polyploidy. This would explain why a small proportion (10–15%) of 2n hepatocytes harbour expanded alleles and that this proportion appears higher in early adulthood than at very old age. In mouse liver, already after few months of age a new end-state of differentiation is reached, and the fraction of multinucleate or polyploid cells does not increase further. Repeat expansion appears to continue over the entire life span of the animal, however (results shown in Table 1 and ref. [13]). When taken together, these observations link expansion most tightly to the post-replicative state, as polyploid cells in liver and pancreas are non-cycling [17-19]. Expansion appears also transcription independent, as the *Dmpk gen*e is expressed at very low levels in hepatocytes and appears fully repressed in acinar cells [[21], own findings, unpublished]. Also other cell types that show polyploidization, like arterial smooth muscle cells, cardiomyocytes and Purkinje cells are generally non-cycling and it will therefore become important to study repeat fate also in these cells. Unfortunately, there are no good experimental protocols available for their isolation, cultivation, and separation into 2n or ≥4n cell pools, so this remains work to be done for the future.

Interestingly, reciprocal correlations exist between the cell state and its DNA recombination-repair capacity that may affect repeat stability. Checkpoint degeneracy with an interesting change in the survey role of p53 has been observed after transition to tetraploidy [22]. We have observed changes in the level of DNA recombination-repair protein RPA, which parallel the process of terminal differentiation and aging of liver (data not shown). A deficiency of DNA repair protein ERCC1 in mice results in accelerated polyploidization rates in liver [23]. Vice versa, polyploidization and associated changes in either cell size, or the process of aging itself [24], may alter cellular metabolism, thereby contributing to changes in the genetic stability of terminally differentiated cells. Metabolic effects on the activities of MLH1 and MutSα, components of the mismatch repair machinery that determine trinucleotide repeat stability, have been reported [25,26] and activity of DNA repair proteins, like Ku, is modulated during aging in liver [27]. Based on these observations and our findings reported here, additional investigations are warranted to understand how changes in both cell nuclearity and DNA ploidy correlate to altered efficacy of the machinery that controls somatic CTG•CAG repeat stability in DM1.

## Methods

### Isolation of liver hepatocytes

Hepatocytes were isolated using a two-step collagenase liver perfusion protocol [20]. After administration of anesthesia and heparin, perfusion was performed via the left ventricle with 30 ml Hepes buffer (25 mM Hepes, pH 7.5, 0.25 mM EGTA), followed by 10 ml Hepes buffer without EGTA, and finally 25 ml Hepes buffer containing 2 mM CaCl_2 _and 0.025% (w/v) collagenase IV (Sigma). After isolation of the liver and disrupting Glisson's capsule, cells were gently pushed out and filtered through a 70 μm filter (Falcon). Three washes with medium (1 × DMEM, 15% FCS, 4 mM glutamine/1 mM pyruvate, 50 μg/ml gentamycin) in combination with low speed centrifugation at 500 rpm for five min were done for hepatocyte enrichment. In our hands, this procedure yielded highly (>95% enriched, but never 100% pure) hepatocyte populations.

### Isolation of pancreatic acinar cells

Mouse pancreatic acinar cells were isolated as described [28,29] with some modifications. Cells were collected after a two-step digestion with Hepes buffer (140 mM NaCl, 5.4 mM KCl, 0.8 mM Na_2_HPO_4_, 25 mM Hepes, 5.8 mM glucose, pH 7.5) including 0.1 mM CaCl_2_, 1 mM MgCl_2 _and 0.05% collagenase IV (Sigma). After filtration through a 70 μm nylon gauze (Falcon), cells were purified through a 4% (w/v) BSA layer in Hepes buffer with 1 mM CaCl_2 _and 1 mM MgCl_2 _by low speed centrifugation (5 min, 500 rpm). Cells were resuspended in Hepes buffer with 1% (w/v) BSA, 1 mM CaCl_2 _and 1 mM MgCl_2_, whereafter the last step was repeated.

### FACS analysis

Single cells were fixed in 70% (v/v) ethanol overnight at -20°C. After a PBS wash, cells were resuspended in PBS containing 20 μg/ml propidium iodide, 0.2 mg/ml RNase and 0.1% Triton X-100, followed by a 15 min incubation at 37°C. Cells were excited at 488 nm and emission was measured at 610 nm using a Beckman Coulter Epics Elite ESP. Diploid, tetraploid and octaploid cells were sorted and isolated. The average number of polyploid hepatocytes in our adult mice ranged between ~50–70% and remained stable after five months of age [17,18].

### PAS staining

To control for purity of our cell population, isolated pancreatic acinar cells were attached on superfrost plus slides and fixed in 4% phosphate-buffered formol (pH 7). Slides were stained for 5 min in periodic acid solution and for 15 min in Schiff solution [30]. Autofluorescence from PAS-positive structures was recorded using a Zeiss Axioplan 2 microscope. Typical preparations obtained, were estimated >99% pure.

### CTG•CAG repeat length analysis

DNA was isolated after proteinase K treatment, phenol/chloroform extraction and isopropanol precipitation. DNA concentrations in different samples were equilibrated in accordance with the number and ploidy of cells used in the sorted population(s) and PCR was performed with forward primer 226 (5'-GAAGGGTCCTTGTAGCCGGGAA-3') and Cy5-labeled reverse primer 225 (5'-GGAGGATGGAACACGGACGG-3') flanking the CTG•CAG tract. PCR products were analyzed on a 6% denaturing acrylamide-urea gel in the ALF DNA sequencing (Amersham Pharmacia) system. PCR products obtained from amplification of Each (CTG•CAG)_n _repeat length profile was normalized to its highest peak to be able to optimally compare the relative contribution of fragments with expanded repeats between different profiles.

## Authors' contributions

WJAAvdB carried out the experiments. DGW designed the figures, supervised parts of the work and helped to draft the manuscript. BW conceived the study and drafted the manuscript. All authors contributed to the design of the study and the analysis and interpretation of the results. All authors read and approved the final manuscript.
